# Efficacy and Safety of Approximately 3 Years of Continuous Ozanimod in Moderately to Severely Active Ulcerative Colitis: Interim Analysis of the True North Open-label Extension

**DOI:** 10.1093/ecco-jcc/jjad146

**Published:** 2023-08-31

**Authors:** Silvio Danese, Remo Panaccione, Maria T Abreu, David T Rubin, Subrata Ghosh, Axel Dignass, Anita Afzali, Douglas C Wolf, Michael V Chiorean, Severine Vermeire, Anjali Jain, Lorna Charles, Garrett Lawlor, Mark T Osterman, Hsiuanlin Wu, James B Canavan, AnnKatrin Petersen, Jean-Frederic Colombel, Miguel Regueiro

**Affiliations:** Department of Gastroenterology and Endoscopy, IRCCS Ospedale San Raffaele and University Vita-Salute San Raffaele, Milan, Italy; Inflammatory Bowel Disease Unit, Gastrointestinal Research, Inflammatory Bowel Disease Clinic, Calgary, AB, Canada; Crohn’s & Colitis Center, University of Miami Miller School of Medicine, Miami, FL, USA; Section of Gastroenterology, Hepatology and Nutrition, University of Chicago Inflammatory Bowel Disease Center, Chicago, IL, USA; University College Cork, Cork, Ireland; Department of Medicine, Agaplesion Markus Hospital, Goethe University, Frankfurt, Germany; University of Cincinnati College of Medicine, Cincinnati, OH, USA; Internal Medicine and Gastroenterology, Atlanta Gastroenterology Associates LLC, Atlanta, GA, USA; Inflammatory Bowel Disease Center, Swedish Gastroenterology, Issaquah, WA, USA; Department of Chronic Diseases & Metabolism, University of Leuven, Leuven, Belgium; Translational Sciences and Medical Affairs, Bristol Myers Squibb, Princeton, NJ, USA; Worldwide Patient Safety, Bristol Myers Squibb, Princeton, NJ, USA; GI Medicine, US Medical Affairs I&F, Bristol Myers Squibb, Princeton, NJ, USA; Disease Area Head, Gastroenterology, Bristol Myers Squibb, Princeton, NJ, USA; Statistician, Bristol Myers Squibb, Princeton, NJ, USA; Bristol Myers Squibb, Princeton, NJ, USA; Clinical Research at Celgene, Bristol Myers Squibb, Princeton, NJ, USA; Department of Medicine, Icahn School of Medicine at Mount Sinai, New York, NY, USA; Digestive Disease Institute, Cleveland Clinic, Cleveland, OH, USA

**Keywords:** Ulcerative colitis, ozanimod, S1P receptor modulator

## Abstract

**Backgrounds and Aims:**

This interim analysis from the True North open-label extension [OLE] study examines efficacy and safety of approximately 3 years of continuous ozanimod treatment in patients with moderately to severely active ulcerative colitis.

**Methods:**

Clinical responders after 52 weeks of ozanimod during the phase 3 True North study, who continued treatment in the OLE, were evaluated. Efficacy, including endoscopic and histological endpoints, was assessed during the OLE for approximately 2 additional years through OLE Week 94, using observed case [OC] and nonresponder imputation [NRI] analyses. Adverse events were monitored from True North baseline through OLE data cutoff and expressed as exposure-adjusted incidence rates.

**Results:**

This analysis included 131 patients; 54% had achieved corticosteroid-free remission at True North Week 52. In OC analyses, clinical response, clinical remission, and corticosteroid-free remission were achieved by 91.4%, 69.1%, and 67.9% of patients, respectively, at OLE Week 94 [146 weeks of total treatment]. Similarly, endoscopic improvement, histological remission, and mucosal healing were achieved by 73.3%, 67.3%, and 56.3% of patients, respectively, at OLE Week 94. Efficacy rates were lower using NRI analyses, but maintenance of efficacy was demonstrated through OLE Week 94. No new safety signals emerged from this analysis. Serious infections, malignancy, cardiovascular events, and hepatic events occurred infrequently.

**Conclusions:**

Among patients who achieved clinical response after 1 year of ozanimod treatment during True North, a high percentage sustained clinical and mucosal efficacy over 2 additional years in the OLE. No new safety signals were observed with long-term ozanimod use.

## Graphical Abstract

**Graphical Abstract:**
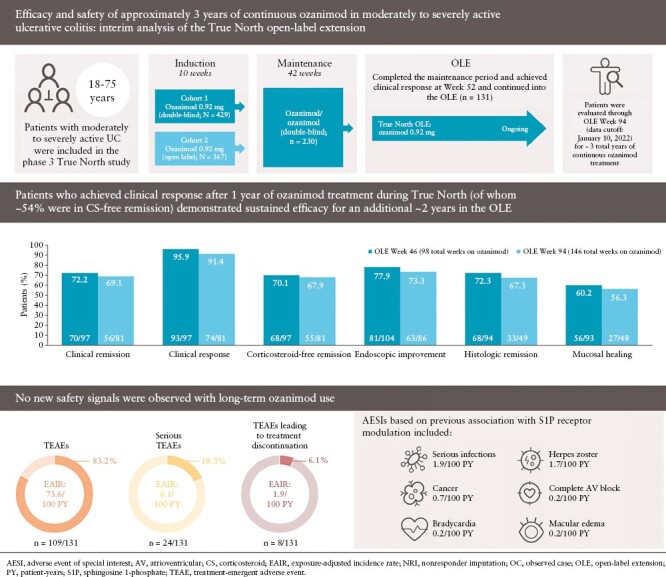

## 1. Introduction

Ulcerative colitis [UC] is an inflammatory bowel disease characterised by a dysregulated immune response and chronic mucosal inflammation of the rectum and colon.^1^ A primary goal of UC treatment is the maintenance of long-term clinical remission, endoscopic healing, and improved quality of life.^2^ Ozanimod is an oral, small molecule, sphingosine 1-phosphate [S1P] receptor modulator that selectively targets the S1P_1_ and S1P_5_ receptor subtypes.^3^ Ozanimod is approved in multiple countries for the treatment of moderately to severely active UC and relapsing forms of multiple sclerosis.^4,5^ While ozanimod recently became the only approved molecule in this novel class for patients with UC,^6^ clinical experience in multiple sclerosis has been reported over 8 years from more than 12 600 patient-years [PY] of treatment.^7^

The phase 3 True North trial evaluated ozanimod 0.92 mg (equivalent to ozanimod hydrochloride [HCl] 1 mg) during a 10-week induction period and a subsequent 42-week maintenance period in patients with moderately to severely active UC.^8^ Treatment with ozanimod resulted in significant improvements in clinical, endoscopic, and histological endpoints compared with placebo, and was well tolerated for up to 52 weeks.^8^ Patients from True North were eligible to enrol in the ongoing True North open-label extension [OLE] trial of ozanimod 0.92 mg.^8^

The aims of this analysis were to provide interim data assessing the long-term efficacy and safety of ozanimod in patients with moderately to severely active UC over approximately 3 years of continuous treatment during the True North study and subsequent OLE. In contrast to many other OLE programmes in inflammatory bowel disease, a distinguishing feature of the True North OLE programme is yearly endoscopic and histological assessment of patients; therefore, this analysis includes these objective data.

## 2. Materials and Methods

### 2.1. Study design

True North [NCT02435992] was a 52-week, randomised, double-blind, placebo-controlled phase 3 trial of ozanimod conducted at 285 sites in 30 countries [Supplementary Figure 1]; the trial design and eligibility criteria have been published previously.^8^ Briefly, patients aged 18–75 years, with moderately to severely active UC, were randomised 2:1 to receive double-blind ozanimod 0.92 mg or placebo in Cohort 1, or received open-label ozanimod 0.92 mg in Cohort 2, for 10 weeks in the induction period. Patients who achieved clinical response to ozanimod at Week 10 were eligible to be re-randomised 1:1 to receive double-blind ozanimod 0.92 mg or placebo in the maintenance period through Week 52; patients with a clinical response to placebo at Week 10 could continue double-blind placebo treatment during the maintenance period.

The True North OLE [NCT02531126] comprised a heterogeneous patient population wherein patients could have entered after True North Week 10 if they did not achieve clinical response during the induction period or from the True North maintenance period after completing Week 52 or upon disease relapse.^8^ Most of the phase 2 TOUCHSTONE OLE study patients were also rolled over into the True North OLE^9^ [Supplementary Figure 1].

Patients who entered the True North OLE from a blinded treatment period [ie, True North induction Cohort 1 and all maintenance groups] initiated OLE ozanimod treatment using the same 7-day dose titration regimen that was implemented at the start of True North.^8^ Patients received ozanimod 0.23 mg [equivalent to ozanimod HCl 0.25 mg] on Days 1–4, ozanimod 0.46 mg [equivalent to ozanimod HCl 0.5 mg] on Days 5–7, and the final dose of ozanimod 0.92 mg starting on Day 8 and continued thereafter. Patients who entered the OLE from an open-label study [TOUCHSTONE OLE] or treatment period [ie, induction Cohort 2] were not required to undergo dose titration and received ozanimod 0.92 mg beginning on Day 1 of the OLE.

The study abides by Good Clinical Practice as described in the International Council for Harmonisation E6 guideline and is being conducted in accordance with the ethical principles outlined in the Declaration of Helsinki and with applicable national, state, and local regulatory requirements. The protocol was reviewed and approved by the institutional review board at each study site. All patients provided written informed consent prior to study entry.

### 2.2. Patient population

This analysis specifically focused on the subset of patients from True North who responded to ozanimod induction therapy, were re-randomised to ozanimod for the maintenance period, and had clinical response at Week 52 before entering the OLE. Clinical response was defined as a reduction from baseline in the 3-component Mayo score of ≥2 points and ≥35% and reduction from baseline in the rectal bleeding subscore [RBS] of ≥1 point or an absolute RBS of ≤1 point. This subset of patients is hereafter referred to as Week 52 clinical responders, as these are patients who received 52 weeks of continuous ozanimod in True North. A subset of the Week 52 clinical responders was also in clinical remission [defined as RBS = 0 and stool frequency subscore [SFS] ≤1 and ≥1 point decrease from baseline SFS and endoscopy score of ≤1]; this subset is hereafter referred to as clinical remitters. Patients were followed for an additional 2 years during the OLE [to at least OLE Week 94], representing at least 146 weeks or approximately 3 years of continuous ozanimod treatment. The True North OLE is ongoing. The data cutoff for this interim analysis was January 10, 2022, at which point all patients had either completed at least 94 weeks of ozanimod treatment during the OLE or discontinued prior to completing OLE Week 94.

### 2.3. Assessments and endpoints

#### 2.3.1. Efficacy

Efficacy was evaluated using the modified Mayo score^10,11^ and individual subscores of Mayo score (ie, RBS, SFS, Physician’s Global Assessment [PGA] subscore, endoscopy subscore). Patients recorded rectal bleeding and stool frequency in daily electronic diaries, and the RBS and SFS were determined at OLE baseline [ie, True North Week 52]; at OLE Weeks 5, 10, 16, and 22; and at every 12-week interval thereafter. The clinician-reported PGA was also collected in electronic diaries. Endoscopy was performed at OLE baseline, at OLE Week 46, and at every 48-week interval thereafter, and was used to determine the endoscopy subscore based on blinded central reading. During endoscopy, two biopsies were taken from the most inflamed area of the left colon and assessed for histological disease activity based on blinded central reading.

Definitions for all efficacy endpoints can be found in Supplementary Table 1. Efficacy endpoints that included an endoscopy and/or histology component were evaluated at OLE Weeks 46 and 94 and included clinical remission, clinical response, corticosteroid-free remission, endoscopic improvement, histological remission, and mucosal healing. Total Mayo score, which included an endoscopy component in addi-tion to the patient- and clinician-reported components, was also evaluated at OLE baseline and OLE Weeks 46 and 94. Efficacy endpoints not including an endoscopy or histology component were assessed at OLE baseline; at OLE Weeks 5, 10, 16, and 22; and at every 12-week interval thereafter through OLE Week 94. These efficacy endpoints included symptomatic clinical response, symptomatic clinical remission, partial Mayo score, and individual Mayo subscores [ie, RBS, SFS, and PGA subscore].

#### 2.3.2. Safety

Adverse events [AEs] were monitored from True North baseline through the OLE cutoff date. Haematology and blood chemistry laboratory measures were assessed throughout the parent trial and at OLE baseline; at OLE Weeks 5, 10, 16, and 22; and at every 12-week interval thereafter. Safety assessments included treatment-emergent adverse events [AEs] [TEAEs], TEAEs leading to treatment discontinuation, serious TEAEs, AEs of special interest [AESIs], and laboratory measures. AESIs were identified based on previous associations with S1P receptor modulation and included bradycardia, heart conduction abnormalities [ie, second-degree and higher atrioventricular block], macular oedema, malignancy, serious or opportunistic infection, pulmonary effects, and hepatic effects. Absolute lymphocyte count [ALC] was evaluated over time at OLE baseline; at OLE Weeks 10, 16, and 22; and at every 12-week interval thereafter through at least OLE Week 94. If ALC was <200 cells/mm^3^, laboratory testing was repeated within 7 days. If confirmed, the study drug was temporarily discontinued and laboratory testing was repeated weekly until ALC was >500 cells/mm^3^, when treatment could be reinitiated.

### 2.4. Statistical analysis

Due to the open-label nature of this study and the lack of a control group, all data were summarised and no formal hypothesis testing was performed. The proportions of patients in symptomatic clinical remission, symptomatic clinical response, clinical remission, clinical response, corticosteroid-free remission, endoscopic improvement, histological remission, and mucosal healing were summarised using observed case [OC] and nonresponder imputation [NRI] analyses. Mean RBS, SFS, PGA subscore, total Mayo score, partial Mayo score, and ALC over time were summarised using OC analyses. NRI analyses evaluated patients who completed each time point or discontinued ozanimod treatment, and missing data were imputed as nonresponses; patients with unavailable histology data at data cutoff were not included in the NRI analyses for the histological remission and mucosal healing endpoints. OC analyses evaluated only the patients with data available at each time point, and missing data were not imputed. The incidences of TEAEs, AESIs, and laboratory assessments were summarised and exposure-adjusted incidence rates [EAIRs] per 100 PY were calculated to adjust for ozanimod exposure on study.

## 3. Results

### 3.1. Patient disposition and characteristics

Of the 457 patients who achieved clinical response while on ozanimod at the end of the True North induction period, 230 were re-randomised to continue ozanimod during the True North maintenance period. A total of 131 patients maintained clinical response through 52 weeks of continuous ozanimod [through end of maintenance] and entered the OLE. Of those 131 Week 52 clinical responders, 83 [63.4%] patients were also in clinical remission at Week 52. At data cutoff, 114/131 patients [87.0%] had completed OLE Week 46 and 94/131 patients [71.8%] had completed OLE Week 94 [Supplementary Figure 2]. At OLE Week 94, 37/131 patients [28.2%] had withdrawn from OLE treatment. The most common primary reasons for withdrawal were patient decision [13/131, 9.9%], lack of efficacy [10/131, 7.6%], and AE [7/131, 5.3%]. Time to discontinuation is shown in Supplementary Figure 3.

Demographics and disease characteristics at True North baseline in the Week 52 clinical responders who entered the OLE are shown in Table 1. Mean age was 44.3 years, and 51.9% of patients were female. Mean duration of UC disease was 8.5 years, and 32.1% of patients had extensive disease. Corticosteroid use at screening was reported in 23.7% of patients, and 68.7% of patients had prior corticosteroid use. Other prior therapy included immunomodulators [35.1%], tumour necrosis factor inhibitors [32.1%], non–tumour necrosis factor inhibitor biologics [19.8%], and any biologics [35.1%; excluding Janus kinase inhibitors]. Baseline demographics and disease characteristics were generally similar for clinical remitters and patients who achieved clinical response without remission at Week 52, as shown in Supplementary Table 2.

**Table 1:** Demographics and disease characteristics at True North baseline of patients who entered the OLE in clinical response.

Characteristic	Week 52 clinical responders^a^ [*n *= 131]
Age, y, mean [SD]	44.3 [13.6]
Female, *n* [%]	68 [51.9]
Body mass index, kg/m^2^, mean [SD]	25.9 [5.8]
Age at UC diagnosis, y, mean [SD]	36.1 [13.4]
Years since UC diagnosis, mean [SD]	8.5 [7.3]
Extent of UC disease, *n* [%]	
Left-sided	89 [67.9]
Extensive	42 [32.1]
Corticosteroid use at screening, *n* [%]	31 [23.7]
Prior therapies, *n* [%]	
5-ASA	129 [98.5]
Corticosteroid	90 [68.7]
Immunomodulator	46 [35.1]
Any biologic^b^	46 [35.1]
TNF inhibitor	42 [32.1]
Non-TNF inhibitor biologic	26 [19.8]

5-ASA, 5-aminosalicylate; OLE, open-label extension; RBS, rectal bleeding subscore; SFS, stool frequency subscore; TNF, tumour necrosis factor; UC, ulcerative colitis.

^a^Clinical response is defined as a reduction from baseline in the 3-component Mayo score [sum of RBS, SFS, and endoscopy subscore] of ≥2 points and ≥35% and reduction from baseline in the RBS of ≥1 point or an absolute RBS of ≤1 point.

^b^Excluding three patients exposed to only Janus kinase inhibitors.

Disease activity at True North baseline [Week 0] and OLE entry [True North Week 52] is shown in Supplementary Table 3. True North baseline Mayo scores, endoscopy subscores, C-reactive protein, and faecal calprotectin were similar for clinical remitters and patients who achieved clinical response without remission at Week 52 and improved from baseline to OLE entry after 52 weeks of continuous ozanimod treatment in all patient groups. At OLE entry, corticosteroid-free remission was achieved by 54.2% of Week 52 clinical responders and by 85.5% of Week 52 clinical remitters.

### 3.2. Efficacy

#### 3.2.1. Symptomatic outcomes over time

**Figure 1:**
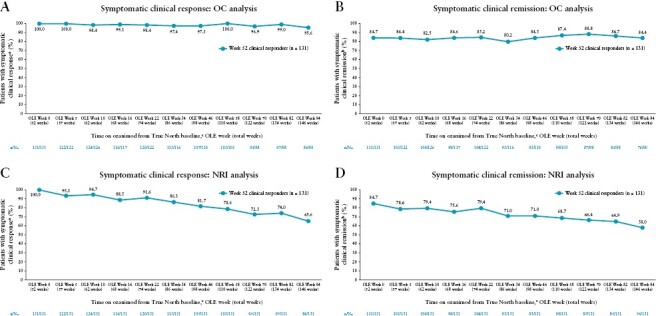
Proportions of patients on continuous ozanimod who entered the OLE in clinical response achieving symptomatic clinical response and symptomatic clinical remission over time in the OLE through OLE Week 94. [A, B] OC analysis. [C, D] NRI analysis. Denominators for the OC analyses were based on the numbers of patients who completed OLE Week 5, 10, 16, 22, 34, 46, 58, 70, 82, or 94 and had data available for the endpoints in question. Denominators for the NRI analyses were based on the numbers of patients who completed OLE Week 5, 10, 16, 22, 34, 46, 58, 70, 82, or 94, or discontinued ozanimod treatment. ^a^Symptomatic clinical response was defined as a decrease from baseline in the combined 6-point RBS + SFS of ≥1 point and ≥30%, and a decrease of ≥1 point in RBS or an absolute RBS ≤1 point. ^b^Symptomatic clinical remission was defined as an RBS = 0 and SFS ≤1, and a decrease of ≥1 point from the baseline SFS. ^c^All patients received 52 weeks of ozanimod treatment before entering the OLE. NRI, nonresponder imputation; OC, observed case; OLE, open-label extension; RBS, rectal bleeding subscore; SFS, stool frequency subscore.

Symptomatic clinical response and symptomatic clinical remission were maintained throughout the OLE. Of the Week 52 clinical responders from True North, 97.3% and 95.6% had maintained symptomatic clinical response after an additional 1 and 2 years of ozanimod at OLE Weeks 46 and 94, respectively, based on OC analyses [Figure 1A]. Symptomatic clinical remission was observed in 84.5% and 84.4% of patients at OLE Weeks 46 and 94, respectively [Figure 1B]. Based on NRI analyses, 81.7% and 65.6% had maintained symptomatic clinical response [Figure 1C] and 71.0% and 58.0% had achieved symptomatic clinical remission [Figure 1D] after an additional 1 and 2 years of ozanimod at OLE Weeks 46 and 94, respectively. Rates of symptomatic clinical response and symptomatic clinical remission were higher in clinical remitters than in patients who achieved clinical response without remission at Week 52, as shown in Supplementary Figure 4.

Reductions in mean total Mayo scores [Supplementary Figure 5A], mean partial Mayo scores [Supplementary Figure 5B], and symptom subscores [ie, RBS, SFS, and PGA] [Supplementary Figure 6] were observed from True North baseline to OLE entry and sustained through OLE Week 94 in the Week 52 clinical responders. During the OLE, mean total Mayo score, partial Mayo score, SFS, and PGA subscore were slightly lower in clinical remitters compared with patients who achieved clinical response without remission at Week 52, whereas RBS was near zero and similar between the subgroups.

#### 3.2.2. Clinical and objective outcomes

**Figure 2:**
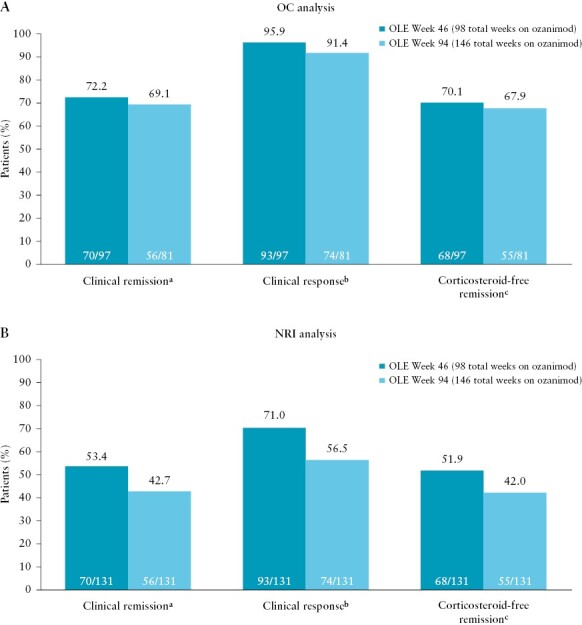
Clinical outcomes [ie, clinical remission, clinical response, and corticosteroid-free remission] at OLE Weeks 46 and 94 in patients on continuous ozanimod who entered the OLE in clinical response. [A] OC analysis. [B] NRI analysis. Denominators for the OC analyses were based on the numbers of patients who completed OLE Week 46 or 94 and had data available for the endpoints in question. Denominators for the NRI analyses were based on the numbers of patients who completed OLE Week 46, completed OLE Week 94, or discontinued ozanimod treatment. ^a^Clinical remission is defined as an RBS = 0 point and an SFS ≤1 point [and a decrease of ≥1 point from the baseline SFS] and an endoscopy subscore ≤1 point. ^b^Clinical response is defined as a reduction from baseline in the 3-component Mayo score [sum of the RBS, SFS, and endoscopy subscore] of ≥2 points and ≥35%, and a reduction from baseline in the RBS of ≥1 point or an absolute RBS of ≤1 point. ^c^Corticosteroid-free remission is defined as clinical remission while off corticosteroids for ≥12 weeks. NRI, nonresponder imputation; OC, observed case; OLE, open-label extension; RBS, rectal bleeding subscore; SFS, stool frequency subscore.

For clinical endpoints in the Week 52 clinical responders from True North, 95.9% and 91.4% of patients maintained clinical response after an additional 1 and 2 years of ozanimod at OLE Weeks 46 and 94, respectively, based on OC analyses [Figure 2A]. Clinical remission was achieved by 72.2% and 69.1% of patients at OLE Weeks 46 and 94, respectively. Notably, rates were similar for corticosteroid-free remission [70.1% and 67.9% at OLE Weeks 46 and 94, respectively]. Based on NRI analyses, 71.0% and 56.5% of patients maintained clinical response, 53.4% and 42.7% achieved clinical remission, and 51.9% and 42.0% maintained corticosteroid-free remission after an additional 1 and 2 years of ozanimod at OLE Weeks 46 and 94, respectively [Figure 2B].

Maintenance of clinical response, clinical remission, and corticosteroid-free remission was higher in clinical remitters than in patients who achieved clinical response without remission at Week 52 in both the OC [Supplementary Figure 7A] and NRI [Supplementary Figure 7B] analyses. Of the patients who achieved clinical response without remission at Week 52, 51.6% were able to achieve clinical remission after another year of ozanimod [OLE Week 46], with 55.6% achieving clinical remission after 2 years of ozanimod [OLE Week 94] in the OC analysis [33.3% and 31.3% at OLE Weeks 46 and 94, respectively, in the NRI analysis].

**Figure 3:**
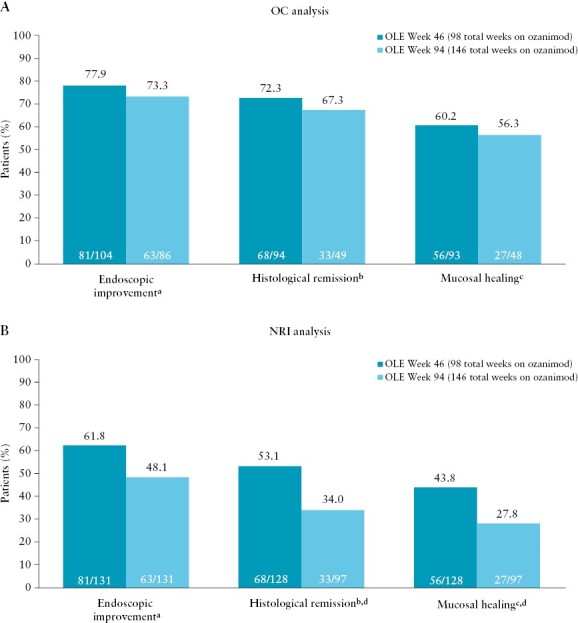
Objective outcomes [ie, endoscopic improvement, mucosal healing, and histological remission] at OLE Weeks 46 and 94 in patients on continuous ozanimod who entered the OLE in clinical response. [A] OC analysis. [B] NRI analysis. Denominators for the OC analyses were based on the numbers of patients who completed OLE Week 46 or 94 and had data available for the endpoints in question. Denominators for the NRI analyses were based on the numbers of patients who completed OLE Week 46, completed OLE Week 94, or discontinued ozanimod treatment. ^a^Endoscopic improvement is defined as an endoscopy subscore of ≤1 point. ^b^Histological remission is defined as a Geboes score of <2.0. ^c^Mucosal healing is defined as an endoscopy score of ≤1 point and a Geboes score of <2.0. ^d^Three patients at OLE Week 46 and 34 patients at OLE Week 94 did not have histology data available at data cutoff and are therefore not included in the denominator for histological remission and mucosal healing. NRI, nonresponder imputation; OC, observed case; OLE, open-label extension.

Endoscopic improvement was achieved by 77.9% and 73.3% of Week 52 clinical responders from True North at OLE Weeks 46 and 94, respectively, based on the OC analysis [Figure 3A]. Rates of histological remission were similar at OLE Weeks 46 and 94 [72.3% and 67.3%, respectively], and rates of mucosal healing were 60.2% and 56.3% at OLE Weeks 46 and 94, respectively. Based on the NRI analysis, 61.8% and 48.1% of patients achieved endoscopic improvement, 53.1% and 34.0% achieved histological remission, and 43.8% and 27.8% achieved mucosal healing after an addi-tional 1 and 2 years of ozanimod at OLE Weeks 46 and 94, respectively [Figure 3B].

During the OLE, achievement of endoscopic improvement, but not histological remission or mucosal healing, was higher in clinical remitters than in patients who achieved clinical response without remission at Week 52 in the OC analysis [Supplementary Figure 8A] and NRI analysis [Supplementary Figure 8B].

### 3.3. Safety

Safety in patients who were on continuous ozanimod during True North and the subsequent OLE for a total of 434 PY of exposure is summarised in Table 2. Rates of TEAEs, serious TEAEs, and TEAEs leading to treatment discontinuation over the ~3-year treatment period [during the True North parent study or the OLE] were 83.2%, 18.3%, and 6.1%, respectively. The most frequently reported TEAEs were lymphopenia [defined as a value that was outside the standard reference range and considered an AE by the investigator], COVID-19 infection, and arthralgia. Serious TEAEs occurring in more than one patient included COVID pneumonia [*n *= 3; 2.3%; 0.7/100 PY], appendicitis [*n* = 2; 1.5%; 0.5/100 PY], and anaemia [*n* = 2; 1.5%; 0.5/100 PY]. Herpes zoster was the only TEAE leading to treatment discontinuation that was reported in more than one patient [*n* = 2; 1.5%; 0.5/100 PY]. During the OLE, one sudden death occurred, of a 57-year-old male patient on study Day 184; the cause and circumstances were unclear, but the event was considered by the investigator as ‘unlikely’ to be related to ozanimod treatment.

**Table 2:** Safety in the True North parent study and the True North OLE in patients on continuous ozanimod who entered the OLE in clinical response.^a^

	Week 52 clinical responders [*n* = 131] Total PY^b^ = 433.9	
	*n* [%]	EAIR per 100 PY^c^
TEAEs	109 [83.2]	73.6
Serious TEAEs	24 [18.3]	6.1
TEAEs leading to treatment discontinuation	8 [6.1]	1.9
Herpes zoster	2 [1.5]	0.5
Lung neoplasm malignant	1 [0.8]	0.2
Lymphopenia	1 [0.8]	0.2
Idiopathic intracranial hypertension	1 [0.8]	0.2
Abdominal pain	1 [0.8]	0.2
Frequent bowel movements^d^	1 [0.8]	0.2
Rectal haemorrhage^d^	1 [0.8]	0.2
Ovarian cyst	1 [0.8]	0.2
Sudden death^e^	1 [0.8]	0.2
Most frequent TEAEs [occurring in ≥5% of patients]^f^		
Lymphopenia^g^	21 [16.0]	5.4
COVID-19^h^	17 [13.0]	4.0
Arthralgia	17 [13.0]	4.3
Hypertension^i^	16 [12.2]	3.9
Headache	15 [11.5]	3.7
Lymphocyte count decreased^g^	14 [10.7]	3.4
Nasopharyngitis	13 [9.9]	3.2
Alanine aminotransferase increased^g^	13 [9.9]	3.2
Anemia^f^	12 [9.2]	3.0
Gamma-glutamyl transferase increased^g^	11 [8.4]	2.7
Back pain	9 [6.9]	2.1
Sinusitis	9 [6.9]	2.2
Upper respiratory tract infection	7 [5.3]	1.7
Herpes zoster	7 [5.3]	1.7
Infection [occurring in ≥3% of patients]^j^	68 [51.9]	24.3
COVID-19	17 [13.0]	4.0
Nasopharyngitis	13 [9.9]	3.2
Sinusitis	9 [6.9]	2.2
Serious infection	8 [6.1]	1.9
Upper respiratory tract infection	7 [5.3]	1.7
Herpes zoster	7 [5.3]	1.7
Respiratory tract infection viral	6 [4.6]	1.4
Bronchitis	6 [4.6]	1.4
Influenza	4 [3.1]	0.9
Gastroenteritis	4 [3.1]	0.9
Malignancies	3 [2.3]	0.7
Basal cell carcinoma	2 [1.5]	0.5
Lung neoplasm malignant	1 [0.8]	0.2
AEs of special interest		
Bradycardia^k^	1 [0.8]	0.2
Complete atrioventricular block^l^	1 [0.8]	0.2
Macular oedema	1 [0.8]	0.2

AE, adverse event; EAIR, exposure-adjusted incidence rate; OLE, open-label extension; PY, patient-years; TEAE, treatment-emergent adverse event.

^a^Data were collected from the beginning of the True North parent study until data cutoff for this analysis [January 10, 2022].

^b^Total PY was defined as the sum of the number of years on study contributed by each patient from time of first dose to last date on study.

^c^EAIRs were calculated as number of patients/PY × 100.

^d^Frequent bowel movements and rectal haemorrhage occurred in the same patient.

^e^The cause and circumstances were unclear; this was considered as ‘unlikely’ to be related to ozanimod.

^f^The most frequent events were defined as those that occurred in ≥5% of patients.

^g^Laboratory values were flagged by the central laboratory if they fell outside the standard reference range; investigators decided whether laboratory values qualified as AEs. Laboratory values that qualified as AEs are reported in this table.

^h^No COVID-19 events occurred during the 52-week True North study, as the study had closed prior to the pandemic.

^i^Of the 16 patients with hypertension, 15 had elevated systolic and/or diastolic blood pressure at baseline and one was normotensive. All cases of hypertension were manageable without a need for ozanimod treatment interruption or discontinuation. All cases of hypertension were nonserious [11/16 were of mild intensity and 5/16 were of moderate intensity], and most [15/16] were considered unrelated to ozanimod.

^j^The most frequent events were defined as those that occurred in ≥3% of patients.

^k^The case of bradycardia occurred on Day 1 of the True North induction period, was considered mild and nonserious, did not require treatment interruption or hospitalisation, and resolved on Day 7.

^l^One patient on ozanimod for 3 years developed an atrioventricular block that was thought to be related to atherosclerotic disease around the cardiac conduction system.

Infections, which most commonly affected the respiratory tract, occurred in 51.9% of patients [24.3/100 PY], with serious infections in 6.1% [1.9/100 PY]. Herpes zoster occurred in 5.3% of patients [1.7/100 PY]. No cases of herpes zoster were serious, few led to treatment discontinuation [*n *= 2; 1.5%], and there were no cases of disseminated herpes zoster. Malignancy occurred in three patients [2.3%; 0.7/100 PY] and included two cases of basal cell carcinoma and one case of malignant lung neoplasm. There was one report of macular oedema [0.8%; 0.2/100 PY], in a patient without comorbidities or other risk factors related to macular oedema. The event was considered mild in intensity and nonserious, and the patient discontinued ozanimod treatment.

There was one report [0.8%; 0.2/100 PY] of bradycardia, which occurred on Day 1 of the True North induction period in a 70-year-old male patient with atherosclerosis. The lowest heart rate reported was 46 bpm 5 h after the first dose of ozanimod. The event was considered mild and nonserious without symptoms or changes in blood pressure, did not require treatment interruption, and resolved on Day 7. Hypertension occurred in 16 patients [12.2%; 3.9/100 PY], with one case of hypertensive heart disease ongoing as of the data cutoff for this analysis [it did not lead to treatment discontinuation and was not considered related to ozanimod treatment]. There was also one report [0.8%; 0.2/100 PY] of complete atrioventricular block, which occurred on OLE Day 766 in a 75-year-old male patient with elevated body mass index and a history of long-standing hypertension. This patient was treated with a permanent pacemaker but did not require ozanimod interruption; the event was considered unrelated to ozanimod treatment, and was instead attributed to atherosclerotic disease affecting cardiac conduction.

Alanine aminotransferase [ALT] levels three or more times the upper limit of normal [ULN] occurred in 11 patients [8.4%], and ALT levels five or more times the ULN occurred in two patients [1.5%]. None of these elevations met the criteria for Hy’s law; most resolved without treatment interruption, but three led to treatment discontinuation.

Patients showed mean reductions in ALC from 1.92 × 10^9^/L at True North baseline to 0.67 × 10^9^/L at OLE entry. These reductions were sustained between 0.72 × 10^9^/L and 0.82 × 10^9^/L through OLE Week 94 [Supplementary Figure 9]. ALC <0.2 × 10^9^/L occurred in 10 patients [7.6%] at any time and most of these reductions resolved without treatment interruption. Only one patient [0.8%; 0.2/100 PY] discontinued treatment because of a TEAE [reported as lymphopenia]; the lowest ALC reported in this patient while on ozanimod was 0.34 × 10^9^/L. There were no reports of serious infection associated with ALC <0.2 × 10^9^/L.

## 4. Discussion

This interim analysis of the phase 3 True North OLE provides evidence for long-term durability and tolerability of approximately 3 years of continuous ozanimod treatment in patients with moderately to severely active UC. In patients who maintained clinical response after 52 weeks of ozanimod treatment in the True North parent study, sustained efficacy was observed for approximately 2 additional years with continued ozanimod treatment through OLE Week 94, based on symptomatic, clinical, endoscopic, and histological measures. Notably, the inclusion of yearly objective endoscopic and histological assessments distinguishes this OLE programme from many other inflammatory bowel disease therapy clinical trial programmes and provides additional objective data to support and interpret these findings. Importantly, no new safety signals were observed with long-term continuous ozanimod use during the True North parent study and the subsequent OLE.

Long-term stability of symptomatic clinical response and remission was maintained by a high percentage of patients throughout the 2-year OLE [through OLE Week 94]. In addition, reductions in mean total and partial Mayo scores, as well as in mean symptom subscores, were observed after 52 weeks of ozanimod in True North and were sustained through OLE Week 94. These findings demonstrate sustained symptom control over approximately 3 years of continuous ozanimod treatment.

Clinical, endoscopic, and histological efficacy endpoints were achieved by a considerable proportion of clinical responders by OLE Week 46, and efficacy rates were generally maintained for another year through OLE Week 94. Of note, more than 90% of patients sustained clinical response. An important goal of UC therapy is corticosteroid-free remission.^12^ In this subset of patients receiving continuous ozanimod treatment for approximately 3 years, 54% of clinical responders achieved corticosteroid-free remission by OLE entry and 70% and 68% of patients maintained corticosteroid-free remission for an additional 1 year [through OLE Week 46] and 2 years [through OLE Week 94], respectively, in the OC analysis. Of patients who were in clinical remission at OLE entry, 86% were in corticosteroid-free remission at OLE entry and 80% and 74% maintained corticosteroid-free remission for an additional 1 and 2 years, respectively, in the OC analysis.

A key strength of the OLE programme for ozanimod in UC is the evaluation of yearly objective endpoints [ie, endoscopic improvement, histological remission, mucosal healing]; such endpoints were not assessed in OLE analyses of many other UC therapies.^13–15^ Achievement of endoscopic and/or histological remission is associated with better long-term outcomes in patients with UC.^2^ In the current analysis of clinical responders, approximately three-fourths of the patients demonstrated endoscopic improvement, more than two-thirds demonstrated histological remission, and more than half demonstrated mucosal healing [which requires both endoscopic improvement and histological remission] after approximately 2 additional years of ozanimod treatment [through OLE Week 94]. These results demonstrated sustained endoscopic and histological benefit for up to 3 years with continuous ozanimod treatment.

Overall, clinical remitters demonstrated nominally greater symptomatic, clinical, endoscopic, and histological benefits with continued ozanimod treatment compared with patients who achieved clinical response without remission at Week 52. These findings indicate that patients who achieve clinical remission in the first year of treatment may have slightly better continued efficacy benefits with long-term ozanimod treatment than those who achieved clinical response without remission. Nevertheless, benefit of continued ozanimod treatment in clinical responders [without remission] was demonstrated, as more than half of these patients went on to achieve clinical remission after an additional year of therapy [at OLE Week 46].

Using the more conservative NRI analysis, efficacy rates were overall lower as compared with the OC analysis, as would be expected. However, clinical response rates were sustained in more than half of the patients at the 3-year visit [OLE Week 94], with nearly half achieving clinical remission and nearly half achieving endoscopic improvement.

This dataset allows us to examine the safety of using ozanimod to treat UC for approximately 3 years. We did not see any new safety signals during the True North parent study^8^ and subsequent OLE. Bradycardia has been associated with S1P receptor modulators and is thought to be mediated by S1P_1_ and S1P_3_ receptor binding in cardiac myocytes.^16^ The risk of bradycardia has been shown to be largely mitigated with gradual dose escalation,^16^ which was implemented over the course of 7 days in True North and the subsequent OLE. The current analysis, representing 434 PY of ozanimod exposure, had only one case of bradycardia, which was asymptomatic, mild, and considered probably attributable to ozanimod. The event occurred on the first day of induction without treatment interruption, and the patient continued on ozanimod for the next 3 years. Other cardiac-related AEs were also infrequent in this analysis. The one case of complete atrioventricular block reported in a patient with elevated body mass index and a long-standing history of hypertension did not lead to treatment discontinuation and was attributed to atherosclerotic disease, not ozanimod. Notably, long-term ozanimod use did not increase the risk of thromboembolic events or major adverse cardiovascular events. There was one sudden death that occurred during the OLE in a patient with a past medical history of myocarditis for which the cause and circumstances were unclear, but the investigator determined that it was ‘unlikely’ to be related to ozanimod treatment.

Immunomodulators, including S1P receptor modulators, may increase the risk of infection or malignancy.^5,17,18^ In this analysis, infections occurred in approximately half of the patients, but the rate of serious infection was low. The incidence rate of herpes zoster was 1.7 per 100 PY, with no serious cases and few leading to treatment discontinuation. The estimated incidence of herpes zoster in the general population of individuals aged ≥45 years ranges from 0.12–2.5 per 100 PY.^19^ Of note, few patients [incidence rate, 0.7/100 PY] developed malignancy in this long-term study. Similar to the finding in the general population, basal cell carcinoma was the most frequent type of malignancy reported in this analysis [incidence rate, 0.5/100 PY]. The estimated incidence of basal cell carcinoma in the general European and US populations ranges from 0.03 to 0.36 per 100 PY.^20–24^ S1P receptor modulators have been associated with an increased risk of macular oedema,^5^ but rates of macular oedema in this analysis were low, with only one occurrence. ALT elevations were also infrequent and mostly resolved without treatment interruption.

Reduction in ALC is an expected pharmacodynamic effect of ozanimod.^5^ In UC and multiple sclerosis clinical trials, the median time for ALC to return to normal range after discontinuing ozanimod was 30 days, with approximately 90% of patients in normal range within 3 months. In the True North parent study, patients who received continuous ozanimod had ALC reductions after 10 weeks of ozanimod induction therapy.^25,26^ These reductions in ALC were sustained for an additional 42 weeks with continued ozanimod treatment in the maintenance period, but began to recover within 8 weeks of discontinuation [time of first follow-up assessment] and returned to pretreatment levels within 18 weeks of discontinuation [time of second follow-up assessment]. The current analysis shows that, with continued ozanimod treatment, ALC reductions were maintained at about the same level for approximately 2 additional years through Week 94 of the OLE. Other analyses from True North confirmed that ALC is a pharmacodynamic marker of ozanimod, but changes in ALC were not associated with ozanimod efficacy, the occurrence of TEAEs or infections, or UC disease activity.^27,28^ Consistently, during True North and the subsequent OLE, ALC levels <0.2 × 10^9^/L occurred infrequently and were not associated with serious infection.

Several limitations of the current analysis are noted. This analysis focused on a subset of the patients that had the maximal ozanimod exposure, resulting in a relatively small sample size. Because this was an open-label study, there was no control group. Patients with less favourable outcomes may have dropped out of the True North parent study, creating a potential selection bias in the subsequent OLE, although this may reflect similarity to real-world treatment selection and continuation.

In conclusion, this interim analysis of the True North OLE demonstrates that patients with moderately to severely active UC, who benefit from ozanimod after 1 year of treatment, have a high likelihood of sustained clinical and mucosal efficacy over approximately 2 additional years of treatment. No new safety signals were identified with long-term ozanimod use; AEs were manageable and infrequently led to treatment discontinuation. The True North OLE is ongoing, and future analyses will provide additional insights into the long-term efficacy and safety of ozanimod for longer periods of time and in larger cohorts of patients.

## Supplementary Material

jjad146_suppl_Supplementary_Material

## Data Availability

Bristol Myers Squibb policy on data sharing may be found at [https://www.bms.com/researchers-and-partners/independent-research/data-sharing-request-process.html].
