# Supplementary material for: Efficacy and Safety of Approximately 3 Years of Continuous Ozanimod in Moderately to Severely Active Ulcerative Colitis: Interim Analysis of the True North Open-label Extension
Source: J Crohns Colitis. 2023 Aug 31;18(2):264–74. doi: 10.1093/ecco-jcc/jjad146 (PMC10896634; doi:10.1093/ecco-jcc/jjad146)
Supplement: jjad146_suppl_Supplementary_Material [file jjad146_suppl_supplementary_material.docx]

**SUPPLEMENTARY MATERIAL**

**Supplementary Figure 1.** True North study design. The purple shading indicates the OLE patient population of interest for this analysis, which comprises patients receiving ozanimod in both the induction and maintenance periods (ozanimod/ozanimod) who completed maintenance in clinical response and entered the OLE (n=131). Patients from the TOUCHSTONE OLE study were not included in this analysis. ^a^Patients stratified by prior tumor necrosis factor inhibitor exposure (yes/no) and corticosteroid use (yes/no) at screening. ^b^Reduction in 3-component Mayo score of ≥2 points and ≥35% from baseline or reduction in 4-component Mayo score of ≥3 points and ≥30% from baseline and reduction in RBS of ≥1 point or absolute RBS of ≤1 point. ^c^Disease relapse was defined as partial Mayo score increase of ≥2 points from Week 10 score or an absolute score ≥4 points, endoscopic subscore of ≥2 points, and exclusion of other causes of an increase in disease activity unrelated to underlying ulcerative colitis. ^d^Includes only patients who completed maintenance with clinical response and subsequently entered the OLE. ^e^At data cutoff, all patients had either completed 94 weeks of ozanimod treatment during the OLE or discontinued prior to completing OLE Week 94. ^f^The total time that patients received continuous ozanimod in True North (52 weeks) and the OLE.

OLE, open-label extension; RBS, rectal bleeding subscore.


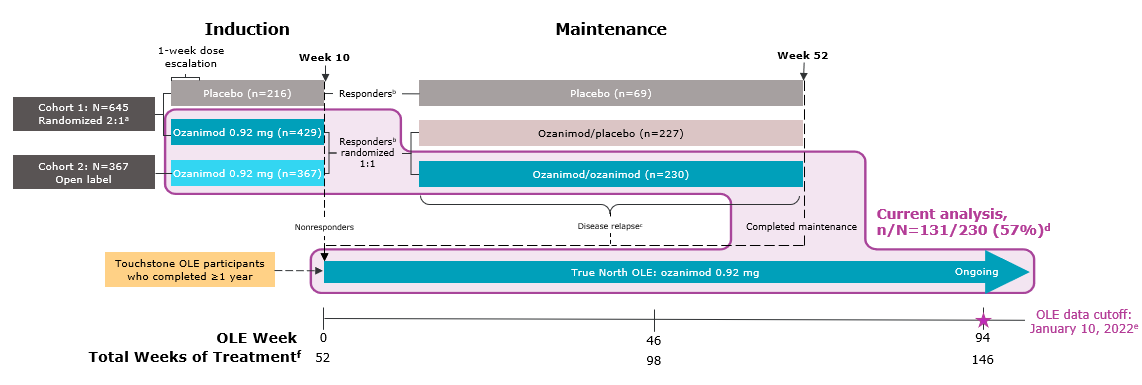
**Supplementary Figure 2.** Disposition of patients who entered the OLE after achieving clinical response following 52 weeks of continuous ozanimod through the True North induction and maintenance periods. ^a^Reason for withdrawal is unknown/unclear. ^b^Reasons for withdrawal were work (n=1), worsening UC (n=1), and unknown/unclear (n=2). ^c^Patient was still symptomatic and needed to switch to another treatment. ^d^Reasons for withdrawal were no benefit (n=1), unavailable to visit site for future study visits (n=1), personal reasons (n=2), no benefit and experienced lymphopenia (n=1), worsening UC (n=1), and unknown/unclear (n=2). ^e^Both patients had lymphopenia. IP, induction period; MP, maintenance period; OLE, open-label extension; UC, ulcerative colitis.


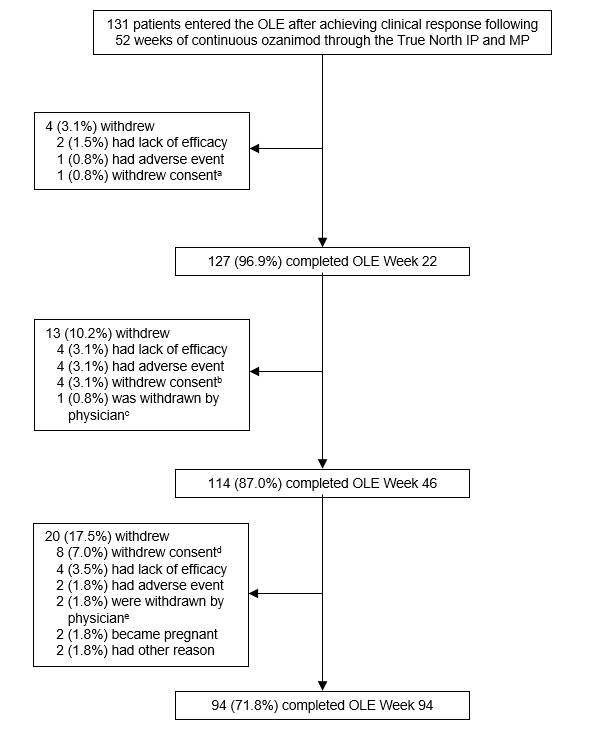


**Supplementary Figure 3.** Time to discontinuation of ozanimod by reason for withdrawal.

**
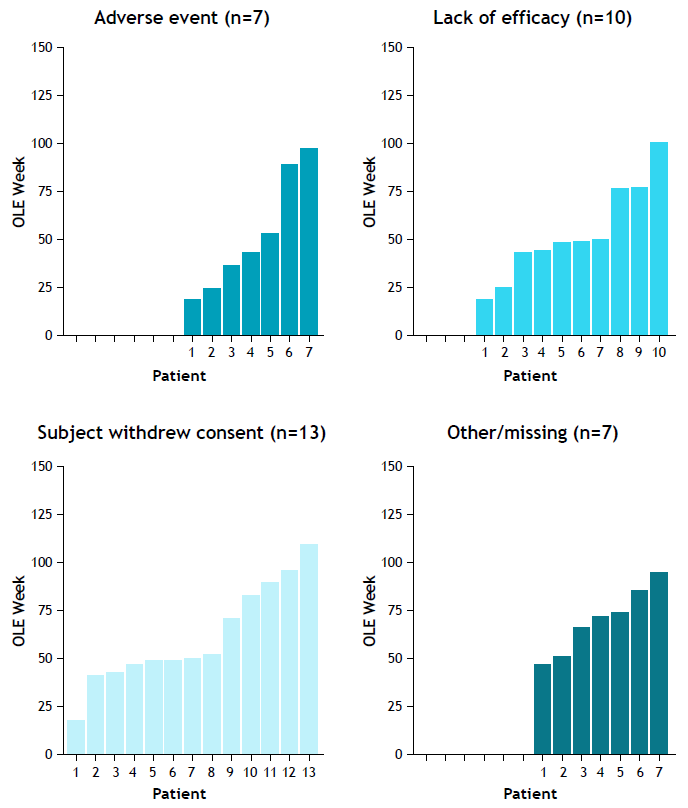
**

**Supplementary Figure 4.** Proportions of patients receiving continuous ozanimod who entered the OLE as clinical remitters or clinical responders (without remission) who achieved symptomatic clinical response and symptomatic clinical remission over time to OLE Week 94. (A, B) OC analysis and (C, D) NRI analysis. Denominators for the OC analyses were based on the numbers of patients who completed OLE Week 5, 10, 16, 22, 34, 46, 58, 70, 82, or 94 and had data available for the endpoints in question. Denominators for the NRI analyses were based on the numbers of patients who completed OLE Week 5, 10, 16, 22, 34, 46, 58, 70, 82, or 94, or discontinued ozanimod treatment. ^a^Symptomatic clinical response was defined as a decrease from baseline in the combined 6-point RBS + SFS of ≥1 point and ≥30%, and a decrease of ≥1 point in RBS or an absolute RBS ≤1 point. ^b^Symptomatic clinical remission was defined as an RBS = 0 and SFS ≤1, and a decrease of ≥1 point from the baseline SFS. ^c^All patients received 52 weeks of ozanimod treatment before entering the OLE. NRI, nonresponder imputation; OC, observed case; OLE, open-label extension; RBS, rectal bleeding subscore; SFS, stool frequency subscore.


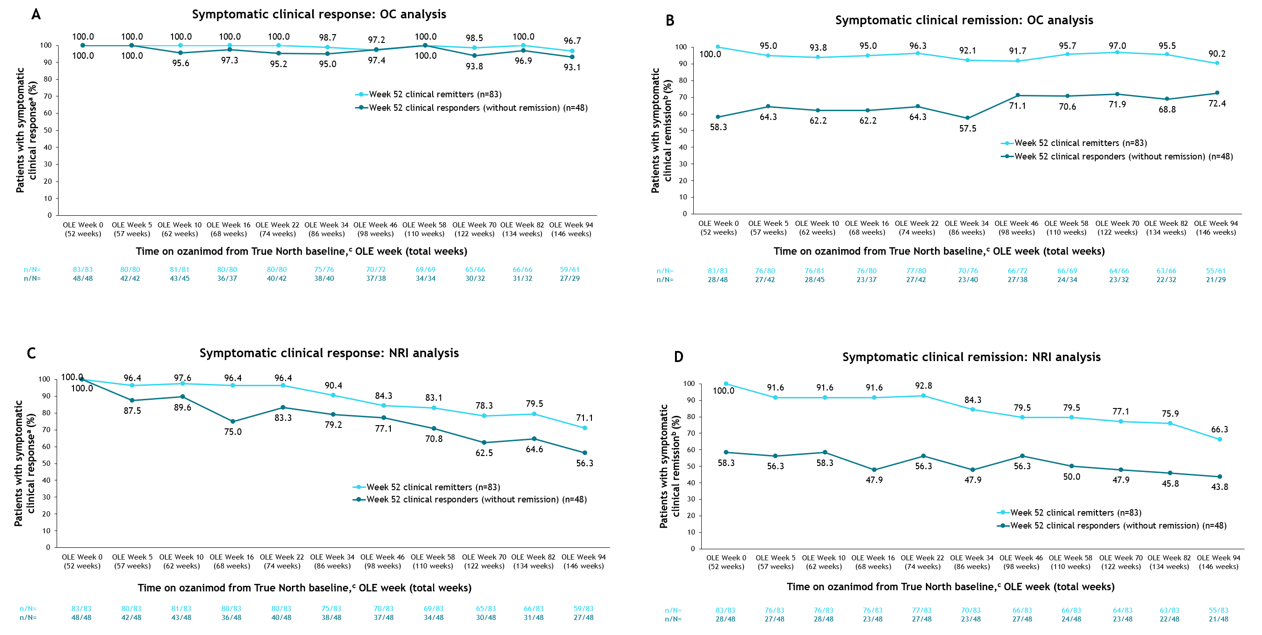


**Supplementary Figure 5.** Reductions in disease activity measured by (A) total and (B) partial Mayo scores over time to OLE Week 94. ^a^Total Mayo score is defined as the sum of the RBS, SFS, PGA subscore, and endoscopy subscore. ^b^Partial Mayo score is defined as the sum of the RBS, SFS, and PGA subscore. ^c^All patients received 52 weeks of ozanimod treatment before entering the OLE. OLE, open-label extension; PGA, Physician’s Global Assessment; RBS, rectal bleeding subscore; SFS, stool frequency subscore; TN, True North.


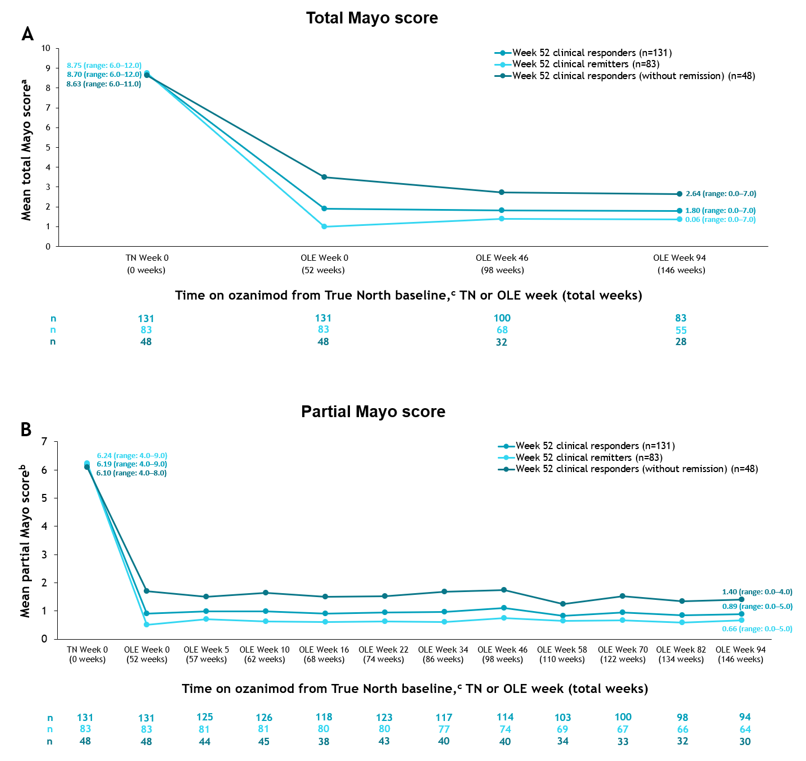


**Supplementary Figure 6.** Reductions in symptomatic disease activity measured by (A) RBS, (B) SFS, and (C) PGA subscore over time in the OLE through Week 94. ^a^All patients received 52 weeks of ozanimod treatment before entering the OLE. OLE, open-label extension; PGA, Physician’s Global Assessment; RBS, rectal bleeding subscore; SFS, stool frequency subscore; TN, True North.


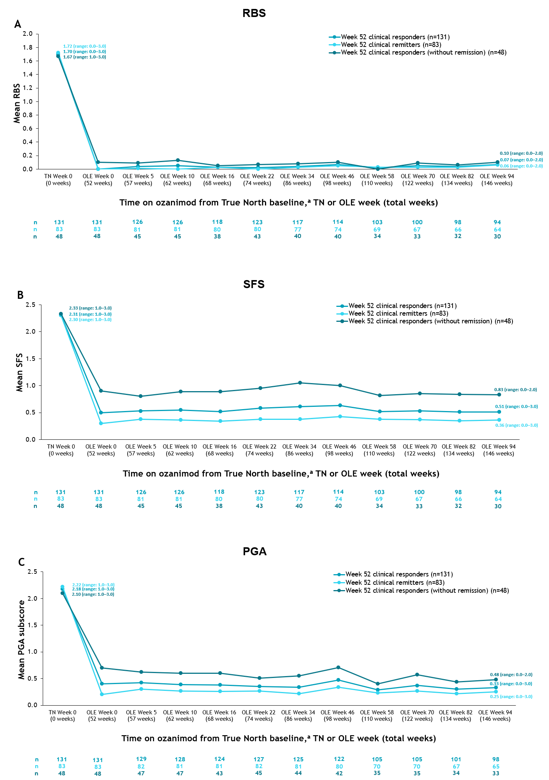


**Supplementary Figure 7.** Clinical outcomes (ie, clinical remission, clinical response, and corticosteroid-free remission) at OLE Weeks 46 and 94 in patients receiving continuous ozanimod who entered the OLE as clinical remitters or clinical responders (without remission). (A) OC analysis. (B) NRI analysis. Denominators for the OC analyses were based on the numbers of patients who completed OLE Week 46 or 94 and had data available for the endpoints in question. Denominators for the NRI analyses were based on the numbers of patients who completed OLE Week 46, completed OLE Week 94, or discontinued ozanimod treatment. ^a^Clinical remission is defined as an RBS=0 point and an SFS ≤1 point (and a decrease of ≥1 point from the baseline SFS) and an endoscopy subscore ≤1 point. ^b^Clinical response is defined as a reduction from baseline in the 3-component Mayo score (sum of the RBS, SFS, and endoscopy subscore) of ≥2 points and ≥35%, and a reduction from baseline in the RBS of ≥1 point or an absolute RBS of ≤1 point. ^c^Corticosteroid-free remission is defined as clinical remission while off corticosteroids for ≥12 weeks. NRI, nonresponder imputation; OC, observed case; OLE, open-label extension; RBS, rectal bleeding subscore; SFS, stool frequency subscore.


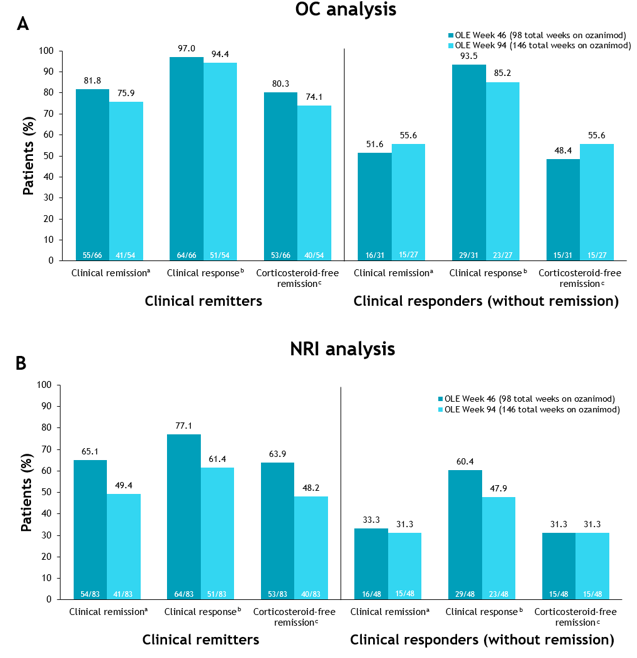
 **Supplementary Figure 8.** Objective outcomes (ie, endoscopic improvement, mucosal healing, and histologic remission) at OLE Weeks 46 and 94 in patients receiving continuous ozanimod who entered the OLE as clinical remitters or clinical responders (without remission). (A) OC analysis. (B) NRI analysis. Denominators for the OC analyses were based on the numbers of patients who completed OLE Week 46 or 94 and had data available for the endpoints in question. Denominators for the NRI analyses were based on the numbers of patients who completed OLE Week 46, completed OLE Week 94, or discontinued ozanimod treatment. ^a^Endoscopic improvement is defined as an endoscopy subscore of ≤1 point. ^b^Histologic remission is defined as a Geboes index score of <2.0. ^c^Mucosal healing is defined as an endoscopy score of ≤1 point and a Geboes index score of <2.0. ^d^Twenty-three patients at OLE Week 94 did not have histology data available at data cutoff and are therefore not included in the denominator for histologic remission and mucosal healing. ^e^Three patients at OLE Week 46 and 11 patients at OLE Week 94 did not have histology data available at data cutoff and are therefore not included in the denominator for histologic remission and mucosal healing. NRI, nonresponder imputation; OC, observed case; OLE, open-label extension.


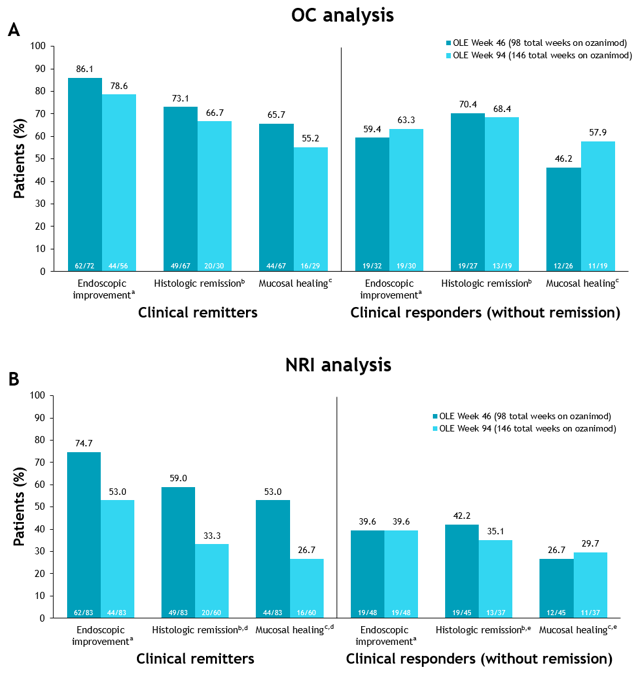


**Supplementary Figure 9.** Absolute lymphocyte count over time to OLE Week 94 in patients on continuous ozanimod who entered the OLE in clinical response. ^a^All patients received 52 weeks of ozanimod treatment before entering the OLE. ALC, absolute lymphocyte count; OLE, open-label extension; TN, True North.


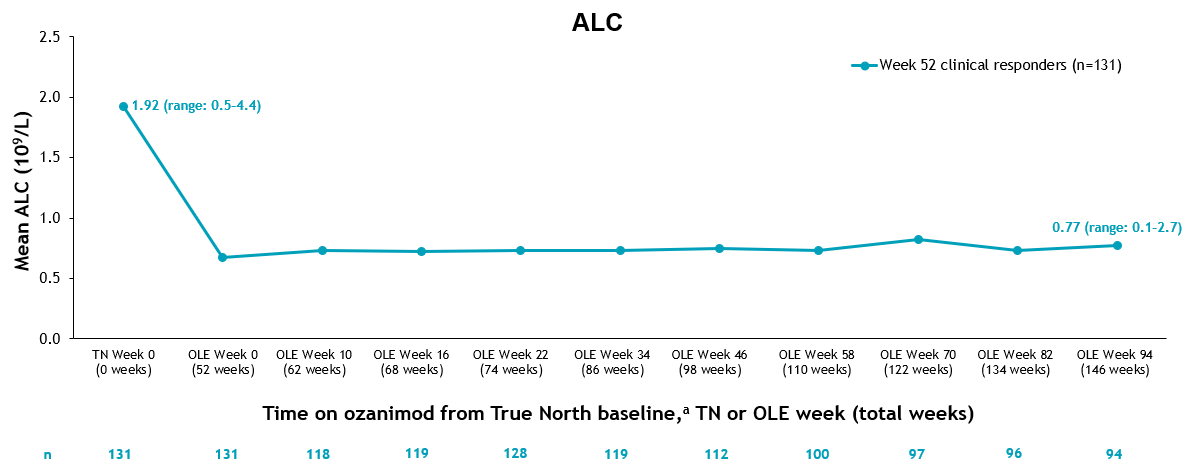


**Supplementary Table 1.** Efficacy endpoint definitions and timing

| **Endpoint** | **Definition** | **Timing of Endpoint Assessment** |
| --- | --- | --- |
| Clinical remission | RBS of 0, SFS ≤1 (and a decrease of ≥1 point from baseline SFS), and endoscopy subscore of ≤1 | OLE Weeks 46 and 94 |
| Clinical response | Reduction from baseline in the 3-component Mayo score (ie, sum of RBS, SFS, and endoscopy subscore) of ≥2 points and 35%, and a reduction from baseline in the RBS of ≥1 point or an absolute RBS of ≤1 point | OLE Weeks 46 and 94 |
| Corticosteroid-free remission | Clinical remission while off corticosteroids for ≥12 weeks | OLE Weeks 46 and 94 |
| Endoscopic improvement | Endoscopy subscore of ≤1 point | OLE Weeks 46 and 94 |
| Histologic remission | Geboes index score of <2.0 | OLE Weeks 46 and 94 |
| Mucosal healing | Endoscopy subscore of ≤1 point and a Geboes index score of <2.0 | OLE Weeks 46 and 94 |
| Symptomatic clinical response | Decrease from baseline in the combined 6-point RBS + SFS of ≥1 point and 30%, and a decrease of ≥1 point in RBS or an absolute RBS ≤1 point | OLE Weeks 0,^a^ 5, 10, 16, 22, 34, 46, 58, 70, 82, and 94 |
| Symptomatic clinical remission | RBS of 0 and SFS of ≤1 (and a decrease of ≥1 point from the baseline SFS) | OLE Weeks 0,^a^ 5, 10, 16, 22, 34, 46, 58, 70, 82, and 94 |
| Total Mayo score | Sum of the RBS, SFS, PGA subscore, and endoscopy subscore (range of 0–12 points) | TN Baseline; OLE Weeks 0,^a^ 46, and 94 |
| Partial Mayo score | Sum of the RBS, SFS, and PGA subscore (range of 0–9 points) | TN Baseline; OLE Weeks 0,^a^ 5, 10, 16, 22, 34, 46, 58, 70, 82, and 94 |

^a^OLE Week 0 is TN Week 52.

OLE, open-label extension; PGA, Physician’s Global Assessment; RBS, rectal bleeding subscore; SFS, stool frequency subscore; TN, True North.

**Supplementary Table 2.** Demographics and disease characteristics at True North baseline of patients on continuous ozanimod who entered the OLE as clinical remitters or clinical responders (without remission)

| **Characteristic** | **Week 52 clinical remitters^a^**  **(n=83)** | **Week 52 clinical responders (without remission)^b^ (n=48)** |
| --- | --- | --- |
| **Age, y, mean (SD)** | 44.2 (12.5) | 44.4 (15.5) |
| **Female, n (%)** | 45 (54.2) | 23 (47.9) |
| **Body mass index, kg/m^2^, mean (SD)** | 25.9 (5.6) | 25.8 (6.2) |
| **Age at UC diagnosis, y, mean (SD)** | 35.5 (12.4) | 37.1 (15.0) |
| **Years since UC diagnosis, mean (SD)** | 9.1 (7.0) | 7.6 (7.7) |
| **Extent of UC disease, n (%)**  Left-sided  Extensive | 55 (66.3)  28 (33.7) | 34 (70.8)  14 (29.2) |
| **Corticosteroid use at screening, n (%)** | 19 (22.9) | 12 (25.0) |
| **Prior therapies, n (%)**  5-ASA  Corticosteroid  Immunomodulator  Any biologic^c^  TNF inhibitor  Non-TNF inhibitor biologic | 81 (97.6)  57 (68.7)  27 (32.5)  22 (26.5)  21 (25.3)  17 (20.5) | 48 (100)  33 (68.8)  19 (39.6)  24 (50.0)  21 (43.8)  9 (18.8) |

^a^Met criteria for clinical remission (defined as RBS = 0 and SFS ≤1 [and a decrease of ≥1 point from baseline SFS] and endoscopy subscore ≤1). ^b^Met criteria for clinical response (defined as a reduction from baseline in the 3-component Mayo score [sum of RBS, SFS, and endoscopy subscore] of ≥2 points and ≥35% and reduction from baseline in the RBS of ≥1 point or an absolute RBS of ≤1 point) but not clinical remission. ^c^Excluding 3 clinical remitter patients exposed to only Janus kinase inhibitors,

ASA, 5-aminosalicylate; OLE, open-label extension; RBS, rectal bleeding subscore; SFS, stool frequency subscore; TNF, tumor necrosis factor; UC, ulcerative colitis.

**Supplementary Table 3.** Disease activity at True North baseline (Week 0) and Week 52 (OLE entry)

| **Characteristic** | **Week 52 clinical responders^a^**  **(n=131)** | | **Clinical responder subgroup: Week 52 clinical remitters^b^**  **(n=83)** | | **Clinical responder subgroup: Week 52 clinical responders (without remission)^c^ (n=48)** | |
| --- | --- | --- | --- | --- | --- | --- |
|  | **TN Week 0^d^** | **TN Week 52^e^** | **TN Week 0^d^** | **TN Week 52^e^** | **TN Week 0^d^** | **TN Week 52^e^** |
| **Total Mayo score,^f^ mean (SD)** | 8.7 (1.5) | 1.9 (1.7) | 8.7 (1.5) | 1.0 (0.9) | 8.6 (1.6) | 3.5 (1.5) |
| **Partial Mayo score,^g^ mean (SD)** | 6.2 (1.3) | 0.9 (1.1) | 6.2 (1.3) | 0.5 (0.7) | 6.1 (1.3) | 1.7 (1.3) |
| **Endoscopy score, mean (SD)** | 2.5 (0.5) | 1.0 (0.9) | 2.5 (0.5) | 0.5 (0.5) | 2.5 (0.5) | 1.8 (0.8) |
| **Endoscopy score, n (%)**  0  1  2  3 | 0  0  64 (48.9)  67 (51.1) | 50 (38.2)  43 (32.8)  31 (23.7)  7 (5.3) | 0  0  41 (49.4)  42 (50.6) | 45 (54.2)  38 (45.8)  0  0 | 0  0  23 (47.9)  25 (52.1) | 5 (10.4)  5 (10.4)  31 (64.6)  7 (14.6) |
| **Corticosteroid-free remission,^h^ n (%)** | 0 | 71 (54.2) | 0 | 71 (85.5) | 0 | 0 |
| **CRP, mg/L, median (IQR)** | 3.0 (1.0, 7.0) | 2.0 (1.0, 4.0) | 3.0 (1.0–8.0) | 2.0 (1.0, 4.0) | 2.0 (1.0–5.5) | 2.0 (1.0, 6.0) |
| **Fecal calprotectin, µg/g, median (IQR)** | 1201 (337.9–2639) | 44.1 (18.1, 252.5) | 1307 (359.3–3008) | 22.7 (14.2, 92.9) | 976.8 (234.8–1675) | 228.1 (67.9, 1126) |

^a^Clinical response is defined as a reduction from baseline in the 3-component Mayo score (sum of RBS, SFS, and endoscopy subscore) of ≥2 points and ≥35% and reduction from baseline in the RBS of ≥1 point or an absolute RBS of ≤1 point. ^b^Clinical remission is defined as RBS = 0 and SFS ≤1 (and a decrease of ≥1 point from baseline SFS) and endoscopy subscore ≤1. ^c^Met criteria for clinical response but not clinical remission. ^d^Baseline of the induction period. ^e^End of the maintenance period/OLE entry. ^f^Sum of the RBS, SFS, PGA subscore, and endoscopy subscore. ^g^Sum of the RBS, SFS, and PGA subscore. ^h^Clinical remission while off corticosteroids for ≥12 weeks.

CRP, C-reactive protein; IQR, interquartile range; OLE, open-label extension; PGA, Physician’s Global Assessment; RBS, rectal bleeding subscore; SD, standard deviation; SFS, stool frequency subscore; TN, True North.
